# Consequences of the emergency response to COVID-19: a whole health care system review in a single city in the United Kingdom

**DOI:** 10.1186/s12873-021-00450-2

**Published:** 2021-05-01

**Authors:** Jeremy W. Tankel, David Ratcliffe, Martin Smith, Andrew Mullarkey, Jennifer Pover, Zoe Marsden, Paula Bennett, Darren Green

**Affiliations:** 1 Salford Clinical Commissioning Group, Salford Civic Centre, Salford, M27 5AW UK; 2 Salford Royal NHS Foundation Trust, Stott Lane, Salford, M6 8HD UK; 3 North West Ambulance Service, Ladybridge Hall, Bolton, BL1 5DD UK; 4 Greater Manchester Health and Social Care Partnership, 3 Piccadilly Place, Manchester, M1 3BN UK; 5 Health Innovation Manchester, City Labs, Nelson Street, Manchester, M13 9NQ UK

**Keywords:** Emergency medicine, General practice, COVID-19, Pandemic, Mortality, Structured judgement review, Response

## Abstract

**Background:**

The response to the COVID-19 pandemic in the United Kingdom included large scale changes to healthcare delivery, without fully understanding the potential for unexpected effects caused by these changes.

The aim was “*to ascertain the characteristics of patients, uncertainty over diagnosis, or features of the emergency response to the pandemic that could be modified to mitigate against future excess deaths”.*

**Methods:**

Review of the entire pathway of care of patients whose death was registered in Salford during the 8 week period of the first wave (primary care, secondary care, 111 and 999 calls) in order to create a single record of healthcare prior to death. An expert panel judged avoidability of death against the National Mortality Case Record Review Programme scale. The panel identified themes using a structured judgement review format.

**Results:**

There were 522 deaths including 197 in hospital, and 190 in care homes. 51% of patients were female, 81% Caucasian, age 79 ± 9 years. Dementia was present in 35%, COVID-19 was cause of death in 44%.

Healthcare contact prior to death was most frequently with primary care (81% of patients). Forty-six patients (9%) had healthcare appointments cancelled (median 1 cancellation, range 1–9). Fewer than half of NHS 111 calls were answered during this period.

18% of deaths contained themes consistent with some degree of avoidability. In people aged ≥75 years who lived at home this was 53%, in care home residents 29% and in patients with learning disability 44% (*n* = 9). Common themes were; delays in patients presenting to care providers (10%), delays in testing (17%), avoidable exposure to COVID-19 (26%), delays in provider response (5%), and sub-optimal care (11%). For avoidability scores of 2 or 3 (indicating more than 50% chance of avoidability), 44% of cases had > 2 themes.

**Conclusions:**

The initial emergency response had unforeseen consequences resulting in late presentation, sub-optimal assessments, and delays in receiving care. Death in more vulnerable groups was more likely to display avoidability themes.

**Supplementary Information:**

The online version contains supplementary material available at 10.1186/s12873-021-00450-2.

## Background

The national response to the COVID-19 pandemic in the United Kingdom included widespread changes to the delivery of healthcare across primary, community, and secondary care, NHS 111, and ambulance services [1, 2]. Changes were implemented at speed without fully understanding the relative impact of COVID-19 versus the potential negative effects of these changes, for instance in the message to Stay at Home for the first week of illness, and an enhanced use of NHS 111 [3]. General Practice (GP) consultations fell by 40% [4], yet the proportion of NHS 111 calls abandoned after waiting > 30 s was 39% in March 2020, compared with 2% for March 2019 [4, 5].

Mortality as a result of the Pandemic is higher in deprived areas. Vulnerable groups are disproportionately affected [6, 7]. Social deprivation is linked to lower health literacy and may therefore generate disproportionate effects from service redesign [8]. Also, patients with learning disabilities may be less able to follow safety netting advice that underpins NHS 111 COVID-19 activity, older patients and those with language barriers and sensory problems may experience challenges in accessing healthcare reliant on technology [9], and not everyone in a deprived inner city has access to a mobile phone with credit [10].

## Method

The established aim of this study of Salford residents who died between weeks 12 and 19 of 2020 (the ‘first wave’ of the COVID-19 pandemic), was to establish whether any phenotypic characteristics of the patients, uncertainty over the diagnosis, or features of the Urgent and Emergency Care (UEC) response to the pandemic could be identified that might be modified to mitigate against excess deaths in similar situations in the future.

All Salford residents registered with a Salford GP and whose death was registered with the Salford Registrar of Birth and Deaths in the 8 week period from 20th March to 18th May) were included. This was the period of excess deaths.

A comprehensive review of all components of care delivered to patients by community, ambulance, and hospital-led services was undertaken by reviewing the computerised records from GPs, secondary care, allied community services, and the 111 and 999 emergency services. This “whole patient journey” approach facilitated understanding of how the changes in care pathways in each domain impacted on the patient.

Salford is the 18th most deprived of the 317 local authority districts in England [11, 12]. In the most deprived parts of the conurbation, life expectancy is 11.9 years lower for men and 8.0 years lower for women than in the least deprived areas.

Records from Hospital, GP, 999 and 111 services were reviewed. GP and Hospital records are directly linked at a patient level to create an integrated Greater Manchester Care Record (GMCR). This too was reviewed. Most patients in care homes in Salford are registered with a single General Practice with joint care from the hospital Care of the Elderly Physicians, facilitating joined-up review of care records. Patient episodes in GMCR and the North West Ambulance Service (NWAS) were reviewed.

Data sharing agreements and a Data Protection Impact Assessment were completed and approved by the Caldicott Guardian for each participating organisation. The approach followed the terms of the NHS Control of Patient Information Regulations 2020 for sharing of information in the context of the COVID-19 pandemic [13]. As such, no formal application for ethical approval was sought, nor was any consent for participation. All data searches, queries and case record reviews were undertaken on computers which hosted secure servers to the hospital, CCG and NWAS records. Patient level data linkage was via NHS number, or by postcode and other features in cases where NHS number was not available (such as 999 calls made by third party bystanders). Patients were allocated a unique ID for this review so that anonymised datasets could be extracted and shared with the review panel as set out below. The study protocol was performed in accordance with the relevant guidelines detailed above.

### Structured judgement review process

A structured judgement review (SJR) approach was adopted [14]. Over 60 data items were extracted relating to patient demographic and co-morbid features, interaction with GP, 111, 999, secondary care, diagnostics, and physiological parameters (as detailed in the results). The reviews covered the period from 1 st March 2020 to the date of death for each patient. A team of clinicians with experience of care quality care reviews from the Health Innovation Manchester Utilisation Management Unit undertook the data collection adopting a mixed methods approach of quantifiable data as well as an initial assessment of clinical themes. Two pilot rounds were undertaken of a total of 50 record reviews to refine the processes and agree on final data extraction terms were undertaken.

A multidisciplinary clinical review panel independently reviewed each SJR and allocated a score using a system aligned to the Royal College of Physicians “avoidability of death” scale from the National Mortality Case Record Review Programme (Table 1). They then met to decide a consensus score for each death [14]. The panel comprised the Medical Director of the Salford CCG, the Urgent Care lead for Greater Manchester Health & Social Care Partnership, the Salford Care Organisation Clinical Director for Mortality, and the Emergency Medicine Consultant representative on the Greater Manchester Mass fatalities and Excess Deaths Planning group. The panel agreed a consensus score as well as any care themes from a predetermined list developed during the pilot phase. The care themes are detailed within the results section. Any single event or theme determined by the panel to be of immediate concern was highlighted to the relevant Care Organisation as part of the review’s governance processes. This was done acknowledging that the reviews undertaken were not comparable to reviews that would be undertaken as part of an organisational serious incident investigation. So the scores detailed here are not presumed to be a definitive measure of actual care delivered. Rather, they are intended to reflect the possible effects of service changes as a result of the pandemic.

**Table 1:** The Royal College of Physicians “avoidability of death” scale

Score 1 Definitely avoidable	
Score 2 Strong evidence of avoidability	
Score 3 Probably avoidable (more than 50:50)	
Score 4 Possibly avoidable, but not very likely (less than 50:50)	
Score 5 Slight evidence of avoidability	
Score 6 Definitely not avoidable	

## Results

### Population characteristics

**Fig. 1:**
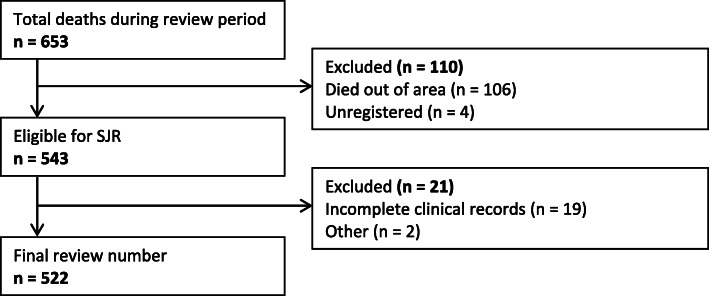
Review exclusion flowchart

Six hundred fifty-three deaths of Salford residents occurred in weeks 12–19 of 2020: 106 died out of area, 4 deaths remained unregistered at the time of the evaluation, 19 had incomplete case records, and 2 could not be accessed in full. There were 522 cases which formed the final review cohort (Fig. 1). The characteristics of these 522 patients are found in Table 2. 51% were female, 35% had dementia, 81% were Caucasian, 9% of mixed or multiple ethnic origin, 10% for other ethnic minority backgrounds, age 79 ± 9 years, 64% cardiovascular co-morbidities. One hundred ninety-seven patients (38%) died in hospital, 190 (37%) in a care home, 120 (21%) at home, and the majority of the remainder in a hospice. Of hospital deaths, 13 died in the Emergency Department (ED) including 5 dead on arrival. Twenty-eight died in a critical care or respiratory high care area.

**Table 2:** Overview of characteristics of the cohort undergoing structured judgement review, divided according to place of death

	Place of death			
	Care home	Own home	Hospital	Other
Number of cases (% of total)	190 (36)	120^a^ (23)	197 (38)	15^b^ (3)
Co-morbid features:				
Age (year, mean ± SD)	85 ± 8	74 ± 14	75 ± 12	69 ± 17
Male gender (%)	38	53	58	60
Diabetes (%)	22	26	25	13
Chronic kidney disease (%)	19	18	22	13
Respiratory (%)	20	40	39	32
Cardiovascular (%)	59	71	66	47
BMI (kg/m^2^, mean ± SD)	22 ± 5	25 ± 8	28 ± 8	27 ± 9
uDNAR or EoL (%)	97	63	71	80
Ethnicity (%):				
Caucasian	81	68	87	93
Asian	0	1	1	0
Black	1	2	3	0
Other BAME	18	29	10	7
Usual residence (%):				
Own home	2	75	53	73
Nursing Home	72	1	9	0
Residential Home	20	0	5	0
Other	6	24	34	27
Medical contact prior to death (%):				
Primary care	96	89	62	80
111	13	10	11	67
999	23	26	86	47
Emergency Department^c^	15	61	19	33
1a listed Cause of death (%):				
COVID-19	54	59	6	13
Acute respiratory, other	23	29	18	7
Acute cardiovascular	2	10	15	0
Cancer	7	11	45	73
Other chronic disease	71	65	42	27
Other acute cause	2	12	5	7
Avoidability of death score (count)				
1	–	–	–	–
2	–	–	2	–
3	2	2	10	–
4	4	7	16	–
5	51	19	56	1
6	133	92^a^	113	14

Key: *SD* standard deviation, *BMI* body mass index, *uDNAR* unified do not resuscitation document, *EoL* end of life. ^a^ this value includes 8 cases of sudden death of unknown cause so no score could be appropriately allocated. ^b^ 14 of 15 were deaths in a hospice. ^c^ indicates hospital visit separate to final admission

Healthcare contact prior to death was most frequently with primary care (81% of patients). Forty-six patients (9%) had healthcare appointments cancelled (median 1 cancellation, range 1–9). There were 60 cases where delays in accessing care occurred.

The directive to prioritise use of the NHS 111 service had unintended adverse consequences. Fewer than half of NHS 111 calls were answered during this period. Twenty-seven of these (46%) resulted in advice to seek further care from their GP or in the community. 9 (15%) resulted in a delay in receiving this care, 5 of whom were seen later in the day by other services and subsequently admitted to hospital.

23% of patients were tested for COVID-19 (*n* = 203). 63% were positive (25% of the review population). The likelihood of a positive swab differed between location of death (68% in care homes, 20% in hospital, 30% in own home). COVID-19 was the most commonly listed cause of death. Eighty-seven of these (43%) did not have a positive swab result. Of the 286 who did not have COVID-19 on the death certificate, 80 (28%) had symptoms consistent with the diagnosis.

### Avoidability of death scoring

80% of cases had an avoidability of death score of 6 allocated by the panel (definitely not avoidable). 18% had a score consistent with some degree of avoidability. 15% had a score of 5 or 4 (slight or possible avoidability). 3% of cases a score of 3 or 2 (more than 50:50 likelihood of being avoidable). None scored 1 (definitely avoidable). 2%, although reviewed for themes, had no final score allocated as these were sudden deaths in a patient’s home with no further information available: the panel did not feel scoring was possible.

**Fig. 2:**
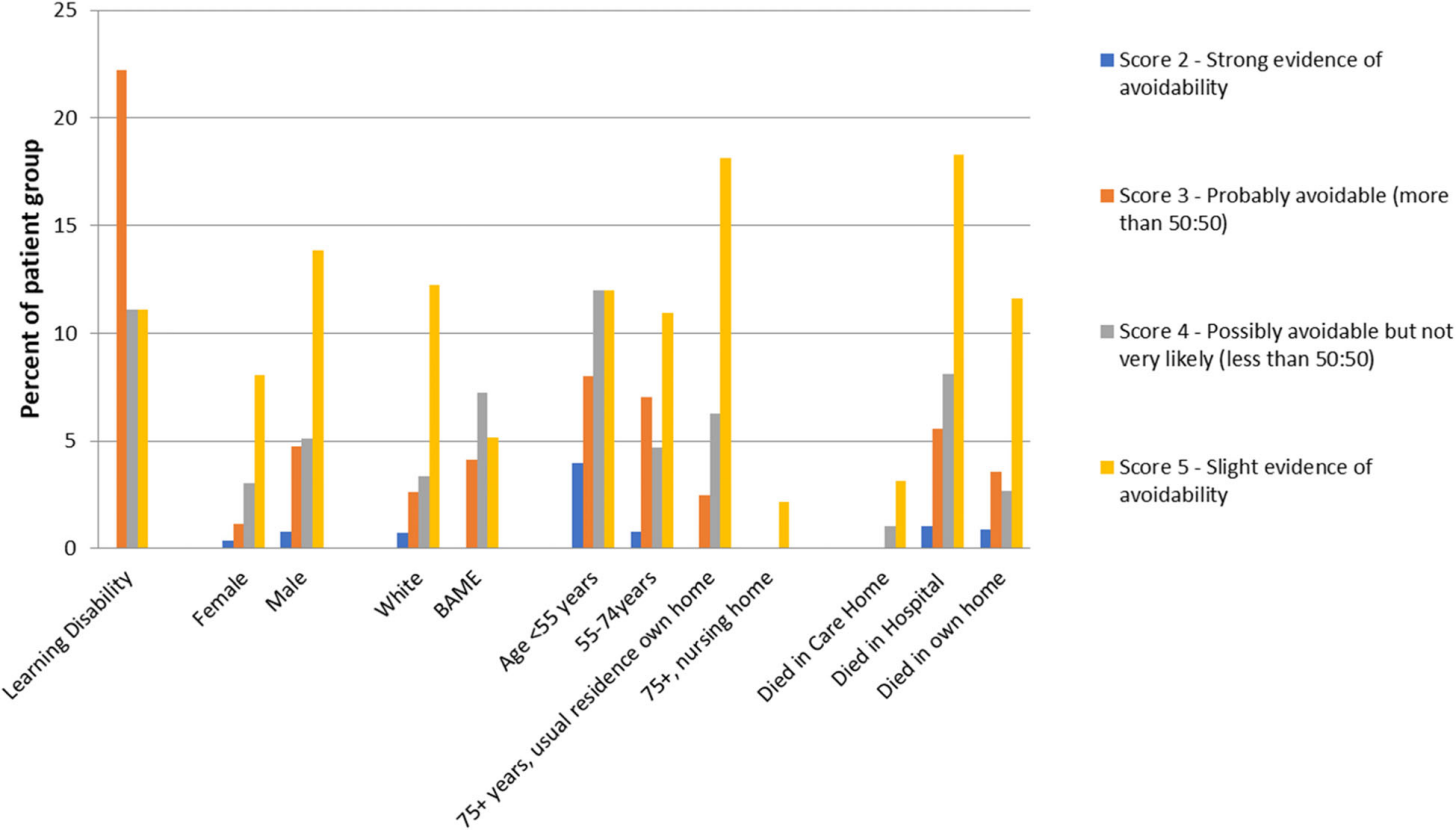
Comparison of distribution of avoidability scores 2 to 5 (indicating findings consistent with some avoidability) between patient groups

The inter-observer agreement of individual reviewer scores was 79%. The free-marginal kappa was 0.75 (95% confidence 0.69–0.80). The fixed marginal kappa was 0.27 (0.12–0.42). Within the context of place of death, the number of cases with at least some potential avoidability were 30% for care home, 23% for death in own home, and 43% for death in hospital. For the age groups < 55 years, 55–75 years, > 75 years the respective figures were 40, 34, and 32%. In older patients, deaths in care homes scored < 6 less frequently than death in other locations (29% versus 53%). This indicates that older patients who resided in their own home were at greater risk of avoidable death than those residing in a care home. For Caucasian patients and non-Caucasian patients the respective figures were 19 and 16%. For COVID-19 and non-COVID-19 deaths the figures were 49 and 23%. The highest frequency of potential avoidability was in patients with learning disabilities (LD), albeit in a small number of deaths (4 of 9 deaths, Fig. 2).

Of the 16 scores of 2 or 3, there were 38 individual examples of recurring themes identified. These were most frequently: delay in accessing care (*n* = 11), below optimal care (*n* = 10), and language barriers (*n* = 3). There was also potentially avoidable COVID-19 contact, delayed response, misdiagnosis, and refusal to attend ED (Table 3).

**Table 3:** Frequency of core themes occurring within the structured judgement reviews, divided according to the overall judgement of avoidability. Patients may have been exposed to more than one theme

Theme	Avoidability score		
	1–3 (> 50% avoidable)	4–5 (< 50% avoidable)	6 (not avoidable)
TOTAL	38	151	461
Below optimal care	10	40	126
Delay in testing	8	32	99
Delay in Accessing care	11	29	20
Capacity Issues (community services)	2	3	3
Exposure to COVID-19	1	19	129
Delay in Response	1	15	16
COVID-19 on MCCD without diagnosis	1	2	47
Communication Barrier	1	2	
Advised to Self-Isolate	1	1	2
Potential Misdiagnosis	1		5
Patient refusal to go to ED	1		
Transfer to IMC no testing		5	3
Sudden Death		1	8
Remote consultation		1	
Self-Discharge		1	
Delay in COVID-19 Swab Results			1
Family Insistence on transfer to ED			1
Hospital Acquired Infection			1

Key: *MCCD* medical certificate of cause of death, *ED* emergency department, *IMC* intermediate care

There was a similar distribution in scores of 4 or 5 (also Table 3) with below optimal care, delays in testing, and delays in accessing care being most common. Themes which occurred relatively more frequently in these cases compared to scores of 2 or 3 were potentially avoidable COVID-19 contact (*n* = 19 of 154 cases) and delayed response (*n* = 15).

Deaths where more than one theme was identified were more common in cases with a greater likelihood of avoidability of death. For scores of 2 or 3, 44% of cases had more than 2 themes. For scores of 4 to 5, 35% of cases had more than 2, and for scores of 6, 17% of cases had more than 2.

### Thematic review

An overview of the 53 patients (9%) showed a delayed presentation to medical services. Within General Practice there were 9 cases of below optimal care, 2 delayed response by clinicians, and 4 examples of lack of service capacity.

For deaths in hospital, there were 255 occasions of themes identified, affecting 129 patients. These included below optimal care in 79 patients, delay in testing 37 patients. Themes most often occurred prior to admission (69% delays in testing, 67% below optimal care, 29 of 30 delays in access). There were 9 hospital deaths after delayed presentation due to patient self-isolation. Four occasions of below optimal care in secondary care were found in patients who were allocated an avoidability score of 2 or 3. These were examples of absence of escalation to higher levels of care.

Residents in care homes were not sufficiently protected from COVID, and isolation from potential contacts with COVID may not have been fully enforced (Table 4). In cases of below optimal care, the majority were issues with Do Not Attempt CPR (uDNAR) directives and Advanced Care (ACP) or Gold Standard Framework (GSF) planning. This is within the broader context of generally excellent end of life planning (92% ACP, 97% uDNAR, and 69% GSF in place at the time of death). There were examples where ambulances were called despite all these being in place.

**Table 4:** Frequency of core contributing factors by major point of care, for all patients and for patients with scores indicating higher likelihood of avoidability. A full breakdown of all factors in all settings is found in supplementary table 1s

	Care Home	111 & 999	GP	Patient	2ndary care
All patients					
Below optimal care	10	2	9	2	32
Delay in testing	17	1	38	4	30
Delay in Access	1	1	0	37	2
Exposure to Covid-19	111	0	0	5	20
Patient (Voluntary Isolation)	0	0	0	10	0
Delay in Response	0	20	2	5	0
No Swabs but COVID-19 on MCCD	0	0	17	0	8
Transfer to IMC no testing	0	0	0	0	6
Score 2, 3 & 4 only (*n* = 39)					
Below optimal care	1	1	3	0	8
Delay in testing	2	1	3	1	6
Delay in Access	1	1	0	13	1
Exposure to Covid-19	1	0	0	2	0
Patient (Voluntary Isolation)	0	0	0	5	0
Delay in Response	0	2	0	1	0

Two hundred ninety-four patients had called 999 (56%). The main theme was delay in response (20 cases). Eleven of these were in patients whose death was considered definitely not avoidable. Fifty-nine were recorded as having called NHS 111 (11%). There were 9 delays in care as a result of 111 calls (15% of calls). Details of the outcomes of all 111 calls and associated themes are found in Table 5. Significant delays in 999 response to Category 1 and 2 calls occurred contributing to avoidable factors in 9 deaths (3%) involving ambulance conveyance, significant delay being a response over three times the 90th Centile (Category 2 90th centile 40 min). The ambulance response took over 2 hours in 44% of Category 2 (immediate and life threatening) cases in March 2020 (mean time 126 min, maximum wait 328 min). The national ambulance response protocol was amended from 2nd April 2020 to mitigate against the emerging pandemic associated delays. This led to a reduction in category 2 delays as detailed in Table 5. Alongside this, the category 1 numbers may contain a number of category 2 ‘upgrades’ i.e. where a response was delayed until the call became critical and then upgraded. This is likely to explain the long dispatch time noted in some category 1 responses. However, it was not possible to differentiate clearly these upgraded calls within the available data. From the beginning of April 2020 no Category 2 patient waited longer than 96 min (mean 44 min, maximum wait 96 min, Table 6).

**Table 5:** Resolution for 111 calls

	N	Themes & comments
Advice only	3	Delays in care for all 3 patients (advised to self-isolate).
Ambulance sent and patient conveyed to hospital	11	
Ambulance Dispatched and patient not taken to hospital	5	All dead on arrival or end of life.
HCP from 111 call back	1	Delay in care.
Clinical assessment service	3	
End of life advice on last day of life	5	4 care home patients in last day of life.
No response from 111	1	Delay of 2 h for 1 patient.
999 response from 111	6	Ambulance dispatch = 5 (4 conveyed to hospital, 3 from care home, 1 delay). No data for 1.
Refer to GP	20	15 care home patients (13 died same day). 3 others referred to hospital by GP. 2 expected deaths at home.
Referral to Pharmacy	1	
Urgent Community Response	3	All 3 delays in care, 2 referred to ED same day by community team.
Total	59

**Table 6:** Ambulance response times divided by those before and after adaptations to the ambulance pandemic response strategy. All times in minutes

	Pre			Post		
	Med ian	IQR	90th cent ile	Med ian	IQR	90th cent ile
Category 1 (*n* = 29 pre, 44 post)						
Ambulance Response Call to Dispatch	4	11	51	3	5	34
Ambulance Response Call to Arrival at Patient	11	23	91	11	14	57
Ambulance Response Arrival at Patient to Arrival at Hospital	46	20	71	45	19	78
Ambulance Response Total time Call to Arrival at Hospital	70	46	115	89	54	220
Category 2 (*n* = 25 pre, 12 post)						
Ambulance Response Call to Dispatch	86	103	205	20	54	71
Ambulance Response Call to Arrival at Patient	99	122	218	35	50	86
Ambulance Response Arrival at Patient to Arrival at Hospital	62	33	85	64	23	78
Ambulance Response Total time Call to Arrival at Hospital	179	110	286	98	74	155
Category 3 (*n* = 4 pre, 7 post)						
Ambulance Response Call to Dispatch	16	61	1245	28	33	150
Ambulance Response Call to Arrival at Patient	26	33	140	49	73	183
Ambulance Response Arrival at Patient to Arrival at Hospital^a^	–	–	–	59	–	–
Ambulance Response Total time Call to Arrival at Hospital^a^	–	–	–	130	–	–

Key: *IQR* interquartile range, ^a^ 1 conveyance to hospital only

For ambulance conveyance to hospital with time from call to arrival < 2 h, 78% of patients had a NEWS2 ≥ 5 on arrival of the paramedic crew to the patient (mean score 7.1 ± 4.0). This compared with 68% for call to arrival 2–4 h (6.3 ± 3.3), and 48% for arrivals > 4 h (4.9 ± 3.6).

An overall summary of the key thematic findings is found in Table 7.

**Table 7:** Summary of key findings

• Many patients delayed seeking care for COVID and non-COVID causes.	
• Delays in ambulance transport could be considerable and may have had an adverse effect.	
• There were unforeseen consequences of the 111 service which resulted in delays.	
• Patients with learning disabilities had the highest likelihood of higher avoidability scores.	
• 1 in 3 deaths occurred in patients with dementia.	
• Older patients in care homes had a low risk of avoidability.	
• Older patients from their own home had a higher risk than younger patients.	
• Below optimal care was the most common theme in cases most likely to be avoidable.	
• There were often long delays in category 2 ambulance responses times early in the pandemic.	
• Avoidability themes were noted in 49% of COVID deaths versus 23% of other deaths.	

## Discussion

This review is the first to use all the documentation in the patient pathway to describe the unintended consequences of the changes that occurred in the emergency response to the COVID-19 pandemic in England and so provides a unique insight.

The national message of *Stay at Home* resulted in delayed presentation of 53 (9%) of patients until very late in their disease course. Whilst this may reflect the unpredictable and often sudden way that COVID-19 can lead to deterioration [15], it is an important factor that may have contributed to delays in care. Fear of COVID-19 (“COVID phobia”) is widely reported and may have stopped some patients seeking help for the illness from which they ultimately died [16]. In the future, patients must be encouraged to seek medical advice when needed and not delay because of the pandemic.

Delays in the response by the ambulance service was evident, and cases where patients were dead on arrival were noted. Ensuring timely transfer of sick patients is postulated to improve survivability but requires further evaluation [17]. The provision of care by NHS 111 delayed detailed assessment of 7 (12%) of the 59 patients who called. The introduction of COVID-19 virtual wards should mitigate this but needs to include all high-risk patients with COVID-19, not just those who have attended hospital [18].

This review has highlighted that older patients (here aged ≥75 years) are more exposed to risk of harm. However, those from care homes had the lowest rate of avoidability themes, which may reflect the safety netting of more immediate access to care. The greater vulnerability of older patients to healthcare associated harm long pre-dates COVID-19 and the current situation serves to highlight this risk [19].

The importance of preventing nosocomial spread of COVID-19 in care homes is again emphasised [20]. Nationally, 13% of hospital COVID-19 infections are by nosocomial spread, and spread through care homes is recognised [21]. This is not fully reflected in this review, where we considered a degree of nosocomial spread to be unavoidable. On an individual basis, it was not possible to state whether more could have been done to prevent infection, except for exceptional cases of missed opportunities for earlier hospital discharge in patients who then went on to contract COVID-19.

A vulnerable patient group may be those with LD. This was recognised prior to COVID-19 through the annual National Learning Disability audit reports. In-patient mortality is 30% in LD patients compared to < 10% in non-LD patients [22]. This review did not note higher risk of avoidability factors in BAME patients, but ethnicity is an important consideration in service reviews following the first wave of the pandemic as noted elsewhere [23]. The higher likelihood of avoidability scoring in male patients appears to reflect gender differences in care homes and that care homes had less avoidability. Regardless, patients at high risk of complications should be contacted as early as possible in their illness and monitored thereafter. Indeed, a low threshold should be set for planned follow up calls or visits for any high risk or vulnerable patient. Where appropriate, this can facilitate early admission to hospital. Early admission is not only important because of the risk of rapid clinical deterioration but also because of potential delays in ambulance response at the peaks of pandemic “waves”.

The theme of below optimal care was common in cases where allocated scores were consistent with potential avoidability. In the community, this was felt often to reflect reliance on remote assessments by the 111 service or primary care. There may be diagnostic limitations not encountered during face to face assessments, especially where there are language barriers. Stay at home advice must be given in the context of the patient’s medical and social vulnerabilities. There is a role for telephone contact for pre-emptive welfare checks for vulnerable relatives of hospitalised positive cases, and a low threshold should be set for planned follow up calls after telephone contact or NHS 111 calls. The importance of effective working links between services to provide joined up care was felt to be lacking early in the pandemic response.

The emerging use of community pulse oximetry and other strategies within the National COVID Oximetry at Home work will provide some safety netting for suspected and confirmed COVID-19 cases, but risk to vulnerable groups less able to access healthcare providers will remain [24]. It must include patients who do not go to hospital as there is evidence that many would benefit from this.

On a narrative level, the panel felt that end of life care was generally excellent. Care homes would benefit from access to additional support at short notice to prevent unnecessary admissions or unnecessary ambulance calls especially in the last stages of end of life. An example of success in this domain is the Electronic Palliative Care Co-ordination System (EPaCCs) which has had success such as increasing the frequency with which patients die in their preferred place [25].

Very high levels of variation in key safety measures within ambulance performance are known to correlate with harm. This is the driver behind the 90th centile approach of the Ambulance Response Programme (ARP) introduced in 2017, to ensure the patients who wait the longest are accounted for [26]. This review demonstrated the validity of this approach with times improving for Q3 and p90 after the implementation of the ‘pandemic protocol’ under the control of the National Ambulance Co-ordination Centre (NACC) in April 2nd 2020. The two key interventions were the implementation of the ‘pandemic protocol’ under the control of the National Ambulance Co-ordination Centre (NACC) and the reduction in demand due to ‘lockdown’. The demonstration of this risk relationship is being used to develop system focussed UEC clinical escalation plans to predict and mitigate harm at times of high demand in Greater Manchester.

Some in-hospital ceiling of care decisions were made that diverged from best practice without a clear documented rationale. It is likely that some escalation decisions were made in the context of an expected overwhelming surge in demand that would outstrip capacity creating a degree of cognitive bias. As evidence of survivability emerges, decisions to not ventilate must be evidence based [27].

## Conclusion

This review took place within a single commissioning catchment area of the UK. Although it was conducted in one of the most deprived conurbations in the UK the themes are felt to be universal and generalisable. A set of core recommendations from themes found during this work are found in Table 8. Ultimately, it is encouraged that whole system reviews of care such as this are undertaken where feasible, and action taken for themes where harm is thought to have occurred. The cases described here are only those where death was the outcome and so it should be expected that the themes that arose are also relevant to and present in the rest of the population with similar characteristics.

**Table 8:** Core recommendations

• Patients must be encouraged to seek medical advice when they have symptoms that they would normally seek advice for, and not delay because of the Pandemic or advice to “Stay at Home”.	
• More targeted face to face assessments may reduce future risk.	
• Stay at home advice must be given in the context of the patient’s medical and social vulnerabilities.	
• Patients at high risk of complications should be contacted early in their illness when medical services become aware of a positive test.	
• Patients with a positive COVID-19 test and symptoms need active reviews to detect early desaturation to facilitate early admission to hospital.	
• A low threshold should be set for planned follow up calls after any telephone contact.	
• Care homes may need access to specialist palliative care at short notice.	
• Pre-emptive telephone welfare checks for vulnerable relatives of positive cases should be considered as they are at high risk of infection.	
• A joined-up approach to follow up between primary care, secondary care, ambulance services, and NHS 111 is necessary after first contact.	
• Pre-emptive discussions about ceiling of care and preferred place of death should be held with care home residents, and families where appropriate.	
• As evidence of survivability emerges, decisions to not ventilate must be fully evidence based.	
• Decision making should be patient focussed, not service focussed.	
• Telephone consultations should not be made with second or third party carers unless unavoidable.	

As a minimum, we recommend that others review the findings of this work in local context, and that a joint consideration of how themes are transferrable to other populations be undertaken. The National Learning from Death Programme highlights the need for robust, transparent review of deaths to ensure that we improve care in a patient focussed manner [28].

## Limitations

This review was not designed to quantify the effect of specific themes, nor to provide a quantifiable risk tool for individual scenarios. It makes no comparison against avoidability or rates of below optimal care prior to the COVID-19 pandemic. The Learning from Death programme and structured judgement review tool, although not designed for use in pandemic situations, ensure that avoidable events that occurred in relation to a death can be addressed and mitigated in a transparent and frank manner. However, because learning from death focusses on events with loss of life as the outcome, it can overshadow the excellent care that most patients receive and the generally favourable outcomes following COVID-19 infection.

The scoring and narrative considerations here were made by an expert panel, introducing the potential for reporting bias based on the expertise and experiences of the panel. Local Authority data was not available via the GMCR at the time of the review. Access to this data may have identified additional features of interest relating to adult social care access and support, especially for vulnerable populations.

## Supplementary Information

**Additional file 1: Supplementary table 1s.** All themes in all settings.

## Data Availability

Selected data from those that support the findings of this study are available from the corresponding author but restrictions apply to the availability of these data, which were used under license for the current study, and so are not publicly available. Data are however available from the authors upon reasonable request and with permission of Salford Clinical Commissioning Group.

## Abbreviations

LD: Learning disability
ED: Emergency Department
NWAS: North West Ambulance Service
CCG: Clinical Commissioning Group
NEWS2: National Early Warning Score 2
uDNAR: Unified Do Not Attempt Resuscitation
GP: General Practitioner
NHS: National Health Service
