# Supplementary material for: Consequences of the emergency response to COVID-19: a whole health care system review in a single city in the United Kingdom
Source: BMC Emerg Med. 2021 May 1;21:55. doi: 10.1186/s12873-021-00450-2 (PMC8087897; doi:10.1186/s12873-021-00450-2)
Supplement: Supplementary file 1 — Additional file 1: Supplementary table 1s. All themes in all settings. [file 12873_2021_450_MOESM1_ESM.docx]

Consequences of the emergency response to COVID-19: a whole health care system review in a single city in the United Kingdom

Jeremy W Tankel FRCGP^1^, David Ratcliffe FRCGP^2,3,4^, Martin Smith FRCEM^2^, Andrew Mullarkey MSc^5^, Jennifer Pover RN^5^, Zoe Marsden BSc^5^, Paula Bennett DProf^5^, Darren Green PhD^2^.

1. *Salford Clinical Commissioning Group, Salford Civic Centre, Salford M27 5AW.*
2. *Salford Royal NHS Foundation Trust, Stott Lane, Salford M6 8HD.*
3. *North West Ambulance Service , Ladybridge Hall, Bolton BL1 5DD.*
4. *Greater Manchester Health and Social Care Partnership, 3 Piccadilly Place, M1 3BN.*
5. *Health Innovation Manchester,* City Labs, Nelson Street, Manchester, M13 9NQ.

Correspondence: Jeremy W Tankel, Medical Director

Salford Clinical Commissioning Group,

Salford Civic Centre, Salford M27 5AW.

[jeremy.tankel@nhs.net](mailto:jeremy.tankel@nhs.net)

0161 983 0190

Supplementary table 1s. All themes in all settings.

|  | Care Home | 111 & 999 | Primary Care EoL | Primary Care GP | Primary Care HCP | Primary Care Patient | Primary care other | Secondary care | Other | Total |
| --- | --- | --- | --- | --- | --- | --- | --- | --- | --- | --- |
| All patients |  |  |  |  |  |  |  |  |  |  |
| All factors | 140 | 24 | 61 | 70 | 17 | 63 | 77 | 112 | 73 | 637 |
| Below optimal care | 10 | 2 | 61 | 9 | 6 | 2 | 10 | 32 | 42 | 174 |
| Delay in testing | 17 | 1 | 0 | 38 | 3 | 4 | 40 | 30 | 6 | 139 |
| Delay in Access | 1 | 1 | 0 | 0 | 1 | 37 | 0 | 2 | 5 | 47 |
| Exposure to Covid-19 | 111 | 0 | 0 | 0 | 0 | 5 | 4 | 20 | 9 | 149 |
| Patient (Voluntary Isolation) | 0 | 0 | 0 | 0 | 0 | 10 | 1 | 0 | 0 | 11 |
| Delay in Response | 0 | 20 | 0 | 2 | 2 | 5 | 0 | 0 | 4 | 33 |
| Capacity Issues | 0 | 0 | 0 | 0 | 4 | 0 | 1 | 1 | 2 | 8 |
| No Swabs but COVID-19 on MCCD | 0 | 0 | 0 | 17 | 0 | 0 | 18 | 8 | 0 | 43 |
| Transfer to IMC no testing | 0 | 0 | 0 | 0 | 1 | 0 | 1 | 6 | 0 | 8 |
| Other | 1 | 0 | 0 | 4 | 0 | 0 | 2 | 13 | 5 | 25 |
| Score 2, 3 & 4 only (n=39) |  |  |  |  |  |  |  |  |  |  |
| All factors | 5 | 5 | 1 | 6 | 3 | 22 | 11 | 21 | 11 | 85 |
| Below optimal care | 1 | 1 | 1 | 3 | 1 | 0 | 3 | 8 | 5 | 23 |
| Delay in testing | 2 | 1 | 0 | 3 | 0 | 1 | 3 | 6 | 2 | 18 |
| Delay in Access | 1 | 1 | 0 | 0 | 0 | 13 | 0 | 1 | 1 | 17 |
| Exposure to Covid-19 | 1 | 0 | 0 | 0 | 0 | 2 | 2 | 0 | 0 | 5 |
| Patient (Voluntary Isolation) | 0 | 0 | 0 | 0 | 0 | 5 | 1 | 0 | 0 | 6 |
| Delay in Response | 0 | 2 | 0 | 0 | 1 | 1 | 0 | 0 | 0 | 4 |
| Capacity Issues | 0 | 0 | 0 | 0 | 1 | 0 | 1 | 0 | 1 | 3 |
| No Swabs but COVID-19 on MCCD | 0 | 0 | 0 | 0 | 0 | 0 | 0 | 2 | 0 | 2 |
| Transfer to IMC no testing | 0 | 0 | 0 | 0 | 0 | 0 | 0 | 1 | 0 | 1 |
| Other | 0 | 0 | 0 | 0 | 0 | 0 | 1 | 3 | 2 | 6 |

Key: MCCD – medical certificate of cause of death; ED = emergency department; IMC = intermediate care, EoL = end of life.
